# Neural mechanisms during role-playing in music psychodrama: an fNIRS Hyperscanning study

**DOI:** 10.3389/fpsyg.2026.1712411

**Published:** 2026-01-30

**Authors:** Ying Wang, Yueqing Zhang, Yuqin Jiang, Yuan Yao, Fupei Zhao, Zhen Zhang, Maoping Zheng

**Affiliations:** 1 Department of Psychology, Southwest University, Chongqing, China; 2 The Communist Youth League Committee of Chongqing Normal University, Chongqing, China; 3 Department of Psychology, Suzhou University of Science and Technology, Suzhou, China

**Keywords:** fNIRS, hyperscanning, inter-brain synchrony, music psychodrama, role-play

## Abstract

The mechanism of inter-brain synchrony (IBS) during role-playing in music psychodrama has received limited empirical attention. To address this gap, the present study employed functional near-infrared spectroscopy (fNIRS) hyperscanning to examine IBS in 46 participant pairs during music psychodrama role-playing. Behavioral results showed that negative emotion questionnaire scores were significantly lower following the intervention compared with pre-intervention levels. Neural results revealed that, relative to the resting state, music psychodrama role-playing significantly enhanced activation in the right dorsolateral prefrontal cortex (R-DLPFC) and the right frontopolar area (R-FT), and also produced a significant increase in IBS within the R-FT. These findings shed light on the neural mechanisms underlying role-playing in music psychodrama and provide empirical support for future intervention research.

## 1 Introduction

Developed by J. L. Moreno, psychodrama is an action-oriented psychotherapeutic approach that employs structured role-playing techniques to help participants gain insight into personal and interpersonal issues while facilitating solution exploration through behavioral interventions. Its dramatic enactments demonstrate not only external behaviors but, more importantly, explore clients’ inner worlds—including unarticulated thoughts and feelings, unexpressed conflicts, and future expectations—thereby creating opportunities for self-reflection (Landis and Skolnik, 2024; Erbay et al., 2018; Sang et al., 2018). Building on his family’s legacy in the field of psychodrama, Moreno (1980) systematically integrated psychodrama with music therapy and formally introduced the concept of musical psychodrama. His 1980 publication, Musical Psychodrama: A New Direction in Music Therapy, is widely recognized as a foundational work that marked the establishment of musical psychodrama as an independent therapeutic modality. This was followed by the influential book Acting Your Inner Music: Music Therapy and Psychodrama, which further solidified the theoretical underpinnings of the field and has since been translated and disseminated in numerous countries and regions worldwide (Moreno, 1999a). Musical psychodrama integrates musical improvisation, music imagery, and other music-based techniques with traditional psychodramatic methods to produce synergistic expressive-arts therapeutic effects. Subsequent studies have summarized the implementation procedures, warm-up techniques, and representative cases of musical psychodrama through direct process observation and qualitative analysis (Moreno, 1984). A consistent theme across these studies is that improvised music can attune to and synchronize with the protagonist’s emotional state, thereby creating a nonverbal support system that acts directly on affective processes, bypasses cognitive defenses, and facilitates deeper emotional expression (Moreno, 1991). Further work in this field has explored the integration of music within expressive arts therapies and highlighted Moreno’s approach as one capable of incorporating multiple art forms. Scholars have also noted that many fundamental components of psychodrama parallel traditional healing practices, situating musical psychodrama within a broader cultural and historical continuum and underscoring its potential to reconnect psychotherapy with its holistic roots (Moreno, 1999b).

The core therapeutic function of music psychodrama lies in combining role-playing with musical improvisation to promote emotional expression and catharsis, enhance self-awareness, and foster cognitive–affective restructuring and creativity. Within this framework, role-playing serves as a central mechanism for achieving these therapeutic outcomes. As a widely applied psychodramatic technique, role-taking (also termed role-play, enactment, or role exchange) has been shown to consolidate knowledge, develop skills and attitudes, and enhance self-awareness strategies (Marco et al., 2009; Nunes et al., 2011). It operates through two key processes: perspective expansion, in which individuals integrate new viewpoints presented during interaction, and perspective alignment, which involves adjusting one’s stance to maintain relational coherence (Puchalska-Wasyl, 2016). These processes support perspective-taking—a cognitive ability essential for generating insights and alternative solutions—and facilitate the development of empathy (Francischetti et al., 2011). In a safe and controlled environment, participants can experiment without real-world risks and benefit from structured opportunities for reflection and feedback (Liberali and Grosseman, 2015). Given that role-playing constitutes the primary mechanism through which music psychodrama exerts its therapeutic effects, examining its underlying cognitive–affective processes provides essential theoretical insights and practical guidance for optimizing therapeutic interventions.

In addition to role-playing, musical improvisation constitutes a core technique of music psychodrama. As a form of musical creativity, improvisation is characterized by spontaneity, flexible yet coherent structures, and continuous interaction with an evolving musical context. Recent research has further clarified why musical improvisation is highly compatible with the spontaneity and creativity emphasized in psychodrama. From a neurocognitive perspective, integrative reviews of electroencephalography (EEG) studies indicate that musical improvisation involves dynamic interactions among cognitive control, emotional processing, and flow-related states. These findings are consistent with the dual-process model of creativity, in which spontaneous and controlled processes are flexibly balanced as a function of individuals’ experience levels (Gnecchi and Antonietti, 2025). From a phenomenological perspective, musical improvisation has been conceptualized as a process of participatory sense-making, in which musical decisions emerge through continuous feedback with the unfolding situation and are guided by a direct, intuitive sense of “rightness.” This process reflects a form of practical rationality rather than highly reflective deliberation (Bergamin, 2024). Taken together, these perspectives support the view of musical improvisation as an embodied, context-sensitive, and interactional mode of action, which closely aligns with psychodramatic processes of scene enactment, role exploration, and interpersonal encounter. Neuroimaging research has shown that engaging in musical improvisation activates core regions of the multiple-demand system, reflecting enhanced reward processing, reduced self-monitoring, and increased socio-emotional flexibility (Liao et al., 2024; Lu et al., 2017). Therapeutically, improvisation has been found to support emotional expression and regulation across diverse populations, facilitating reward engagement in children without musical training and fostering creative self-expression and psychosocial well-being in individuals with early-onset dementia (Barrett et al., 2025; Dowlen et al., 2024). Its accessibility to non-musicians further highlights its suitability for therapeutic and research contexts involving the general population.

Despite extensive qualitative and clinical descriptions of music psychodrama, its underlying neural mechanisms—particularly those involving interpersonal coordination and affective resonance—remain largely unexplored. This gap calls for neuroscientific approaches capable of capturing real-time socio-emotional processes during interactive therapeutic practices. Functional near-infrared spectroscopy (fNIRS) offers strong ecological adaptability, enabling stable recording of cortical activity—particularly within the prefrontal regions—during highly interactive and naturalistic settings. This makes it well suited for investigating music psychodrama, a therapeutic approach that relies heavily on real-time interpersonal engagement (Czeszumski et al., 2020). Meanwhile, interpersonal coordination and affective resonance are central mechanisms in music psychodrama, and inter-brain synchronization (IBS) provides a direct quantitative index for assessing such neural coupling during dyadic interaction. Previous studies have shown that stronger IBS is often associated with higher levels of cooperation and emotional coordination, providing critical support for examining interpersonal neural dynamics in therapeutic contexts (Li et al., 2015; Wang S. K. et al., 2025). Building on these advantages, combining fNIRS with IBS analysis enables both high ecological validity and precise characterization of key interpersonal processes. On the one hand, fNIRS allows for stable acquisition of brain activity in naturally interactive music-psychodrama settings. On the other hand, IBS objectively reflects affective resonance, co-regulation, and collaborative engagement between participants. Moreover, core mechanisms implicated in music psychodrama—such as mentalizing, empathy, and emotion regulation—are primarily supported by cortical networks in the prefrontal cortex and temporoparietal junction, which can be effectively targeted by fNIRS to examine their functional contributions. Therefore, this integrative methodological approach provides a robust framework for elucidating the neural underpinnings of music psychodrama (Rojiani et al., 2018).

Given that fNIRS and IBS can capture the real-time interactive mechanisms involved in music psychodrama, it is necessary to further elucidate its mechanisms of action from a theoretical perspective. The following three theoretical frameworks provide essential support for understanding its therapeutic effects. Role theory, originating from Mead’s (1934/2002) analysis of “taking the role of the other,” posits that understanding oneself requires adopting the viewpoint of another—whether a concrete or generalized other. Blumer (1969/1991) further emphasized that social roles are continually shaped and revised through interaction and reflexive interpretation (Mead, 1934/2002). Adopting another’s perspective requires temporarily suspending habitual cognitive patterns, while enacting a role elicits immediate verbal, behavioral, and emotional feedback from interaction partners. These processes facilitate the integration of self–other representations, promote flexible emotion regulation, and support relational reconstruction. This theoretical foundation underlies the use of role-based techniques in music psychodrama and explains how role-playing contributes to therapeutic change.

Psychodynamic psychotherapy centers on exploring unconscious aspects of the self that emerge within relational contexts (Shedler, 2010). The spontaneity and creativity inherent in musical psychodrama align closely with psychodynamic principles. Moreno (1955) conceptualized spontaneity as the readiness that initiates a creative process, with creativity constituting the final product. Through nonverbal emotional expression, reduced defenses, symbolic enactment, and relational interaction, musical improvisation provides access to unconscious affective states and internalized relational patterns, thereby supporting psychodynamic mechanisms of change (Moreno, 1991; Rojiani et al., 2018).

Empathy-based models—including affective empathy, cognitive empathy, and participatory sense-making—emphasize that individuals’ emotional and cognitive states can be shared and coordinated through dynamic interaction, and that role-playing offers a viable avenue for probing embodied empathy (Sletvold, 2015). Empathy research shows that affective resonance between partners is associated with synchronized activation in regions involved in social cognition and emotion regulation, such as the prefrontal cortex, temporoparietal junction (Balconi and Vanutelli, 2017; Wang et al., 2022). During musical improvisation and role-playing, partners continuously predict, infer, and adjust to each other’s actions and psychological states, forming a co-regulatory process that constitutes the neural basis of empathy. In summary, by integrating insights from role theory, psychodynamic theory, and empathy-based interpersonal frameworks, this study constructs a coherent “music–action–drama” integrative model for understanding the mechanisms of musical psychodrama. This theoretical synthesis not only enriches the expressive arts therapy literature but also provides a scientific foundation for clinical applications, offering meaningful implications for evidence-based practice and mental health promotion.

Although existing studies have consistently shown that psychodrama can effectively reduce negative emotions through mechanisms such as emotional expression, role reversal, and interpersonal support, the specific mechanisms by which musical psychodrama influences negative affect remain insufficiently explored (Wang et al., 2020; Giacomucci et al., 2025; Grigorescu et al., 2020). Building on this foundation, findings from studies on role-playing and musical improvisation offer important insights into the therapeutic processes by which musical psychodrama may reduce negative emotional states. One study indicated that role-playing facilitates decentering, enabling individuals to create psychological distance from stressful or traumatic experiences and to achieve emotional release—such as reductions in anxiety and depression—through physical movement and immersive role enactment (Singla et al., 2023). Another study identified four distinct mechanisms underlying musical improvisation, one of which is its ability to communicate complex or suppressed emotions without relying on verbal expression (MacDonald and Wilson, 2014). For example, Raglio et al. (2016) found that integrating free improvisation–based music therapy with speech and language therapy produced better outcomes in aphasia rehabilitation than speech and language therapy alone. Furthermore, research on one of the core techniques of musical psychodrama—the doubling technique—provides additional supporting evidence. A meta-analysis of 25 psychodrama studies revealed that the doubling technique yielded the strongest therapeutic effects. This technique can function as an auxiliary ego, mirroring the protagonist’s inner experiences and accessing private emotional imagery, or as an extension of the protagonist, articulating subconscious or avoided emotions to foster awareness and action (Kipper and Ritchie, 2003). Another study further reported that the therapeutic effects of the doubling technique follow a dynamic temporal trajectory: initially facilitating emotional expression by reducing defensive mechanisms, and subsequently evolving into enhanced and sustained participant engagement throughout the intervention (Goldstein, 1967). Taken together, these findings suggest that integrating role-playing, musical improvisation, and the doubling technique within musical psychodrama may promote emotional release and self-regulation through experiential interaction. Accordingly, the present study introduces an innovative paradigm—improvisational role-playing incorporating the doubling technique—to investigate its underlying mechanisms.

Neuroimaging research has progressively elucidated the neural mechanisms underlying role-playing in psychodrama. fNIRS studies have demonstrated activation of the prefrontal cortex during psychodramatic performance (Lim et al., 2024). The study by Ames et al. (2008) further corroborated the well-established association between empathy and role-playing. An fMRI investigation on perspective-taking revealed enhanced recruitment of self-mentalization networks, including the ventromedial prefrontal cortex, when participants were instructed to adopt a first-person perspective (i.e., “imagine you are person X”) compared to a third-person perspective. These findings were interpreted as reflecting enhanced empathy during perspective-taking, characterized by a subjective “blurring” of self-other boundaries (Ames et al., 2008). Majdandžić et al. (2016) further reported that mentalizing, which involves reflecting on the thoughts, beliefs, and emotions of others, can enhance subsequent empathy and prosocial behavior. During this process, activation in the dorsomedial prefrontal cortex was found to predict the intensity of prosocial behavior. Additionally, Neuroimaging evidence indicates that improvisation enhances functional connectivity within emotion- and reward-related regions while modulating key areas such as the prefrontal cortex (PFC) and temporoparietal junction (TPJ) —brain regions critically involved in executive function, social cognition, and perspective-taking (Limb and Braun, 2008; Beaty, 2015; Sasaki et al., 2019). Because musical improvisation in psychodrama—unlike verbal improvisation—primarily engages right-hemisphere regions involved in emotion, the R-PFC (the right prefrontal cortex) and R-TPJ (the right temporoparietal junction) have emerged as key regions of interest for exploring the neurocognitive mechanisms of role-playing in naturalistic contexts (Zhang et al., 2020; Xu et al., 2023; Rojiani et al., 2018). Despite these informative references, empirical research on the mechanisms and neural correlates of musical psychodrama remains scarce, with virtually no experimental studies reported to date. To address this critical gap, the present study aims to investigate the neural substrates of musical psychodrama, with the objective of elucidating its underlying mechanisms and providing empirical support for its clinical applications.

This study employs fNIRS-based hyperscanning to examine the behavioral and neural mechanisms underlying role-playing in music psychodrama. The hypotheses are as follows:

*Hypothesis 1*: Music psychodrama grounded in role-playing will effectively reduce negative emotional states in university students with social anxiety, as reflected by decreased negative affect scores on standardized measures such as the Positive and Negative Affect Schedule (PANAS). In this way, music psychodrama is expected to function as a form of emotional regulation and psychological support.

*Hypothesis 2*: Building on established fNIRS paradigms used in psychodrama research, the present study further hypothesizes that, compared with the resting state, the incorporation of the doubling and improvisation techniques during role-playing will significantly enhance intra-brain activation and IBS in the R-PFC and R-TPJ.

## 2 Methods

### 2.1 Participants

An *a priori* power calculation was performed in G*Power (v3.1.9.7). Assuming 95% power, an alpha level of 0.05, and a medium target effect (Cohen’s *f* = 0.25) for a within-participant design, the analysis indicated that 84 individuals (i.e., 42 dyads) would be sufficient. Given the possibility of channel dropouts and suboptimal signal quality during fNIRS preprocessing, we recruited 46 dyads characterized by social-anxiety traits through convenience sampling (*N* = 92). The sample comprised 36 female–female pairs and 10 female–male pairs, with ages ranging from 18 to 28 years (*M* = 20.13 years).

Trait social anxiety was evaluated using the Interaction Anxiousness Scale (IAS) following the Manual of Mental Health Rating Scales (Revised Edition). All participants obtained IAS scores of 39 or higher (possible range: 15–75), satisfying the inclusion criterion for a social-anxiety-trait cohort. The IAS (Leary, 1983) includes 15 items rated on a 5-point Likert scale (1 = “not at all characteristic of me,” 5 = “extremely characteristic of me”) and shows robust psychometric performance, with an 8-week test–retest reliability around 0.80, item–total correlations exceeding 0.45, and Cronbach’s *α* above 0.87 (Wang Y. et al., 2025; Leary, 1983).

We focused on individuals with social-anxiety traits for two considerations. First, such individuals typically display amplified affective and physiological sensitivity during social exchange, which increases the detectability of intervention-related behavioral and neural modulation (e.g., during role reversal). Second, using a relatively homogeneous trait group reduces baseline variability, allowing observed IBS changes to be more confidently linked to experimental manipulation. Role reversal directly addresses hallmark features of social anxiety, including inflexible self-representations and reduced empathic capacity. More broadly, by integrating psychodrama with neuroscience, the study seeks to generate neurobiological evidence and theoretical insights relevant to psychotherapeutic approaches for social anxiety. Although this sampling strategy may constrain immediate generalization, it offers a necessary theoretical platform and preliminary empirical basis for future testing in more diverse populations.

All participants reported right-handedness, normal or corrected-to-normal vision, and no history of neurological or psychiatric illness. Before data collection, they completed ~30 min of standardized instruction in basic psychodrama procedures and musical improvisation to ensure task familiarity. The protocol was approved by the Ethics Committee of Southwest University (Approval No. H25091; March 7, 2025). Written informed consent was obtained, and participants received ¥50 RMB after completing the experiment.

### 2.2 Experimental tasks and procedure

The study was carried out in a quiet, sound-insulated laboratory. Ambient noise was kept below 40 dB, and lighting and temperature were maintained at standard, comfortable levels. All sessions were video-recorded throughout to enable subsequent behavioral coding. Because of their simplicity and their capacity to express affect through rhythmic features, drums and tambourines were chosen for musical improvisation. To maximize ecological validity and allow spontaneous interaction, participants were not restricted with respect to tempo or rhythmic patterns. Following the conventional music-psychodrama workflow, the resting (silent sitting) and role-playing conditions were administered in a fixed sequence without randomization.

The intervention was jointly implemented by two doubles who had received standardized, study-specific training prior to the experiment. Both doubles participated in approximately 5 days of intensive training and practical rehearsal before data collection, which was primarily conducted by a teacher with extensive experience in psychodrama practice. The training covered the basic principles and core techniques of psychodrama (i.e., role reversal, doubling, and mirroring) and placed particular emphasis on task-specific, scenario-based practice aligned with the experimental paradigm (e.g., consistency in role enactment during dyadic interaction, standardization of prompts and interaction tempo, and strategies for managing and stabilizing emotional responses), thereby ensuring procedural uniformity and reproducibility of the experimental protocol. In addition, a professionally qualified music therapist participated in the training and provided assistance and supervision during the experimental implementation, with particular attention to the application of musical improvisation for emotional expression and interpersonal interaction. Throughout the training and data collection process, supervision focused on maintaining intervention consistency, emotional holding/containment, and procedural fidelity, in order to ensure the stability and reproducibility of the intervention procedures employed in this study.

During the protocol, role-playing functioned as the primary phase for scenario enactment and for establishing an interactional baseline. Participants engaged in face-to-face musical improvisation after entering either their own roles or roles assigned by the procedure. Given that individuals with social-anxiety traits may be less comfortable with verbal expression, the doubles mainly offered verbal scaffolding and emotional support, and joined the instrumental exchange only when additional affective elicitation was needed, thereby preserving the continuity and naturalness of interaction.

**Figure 1:**
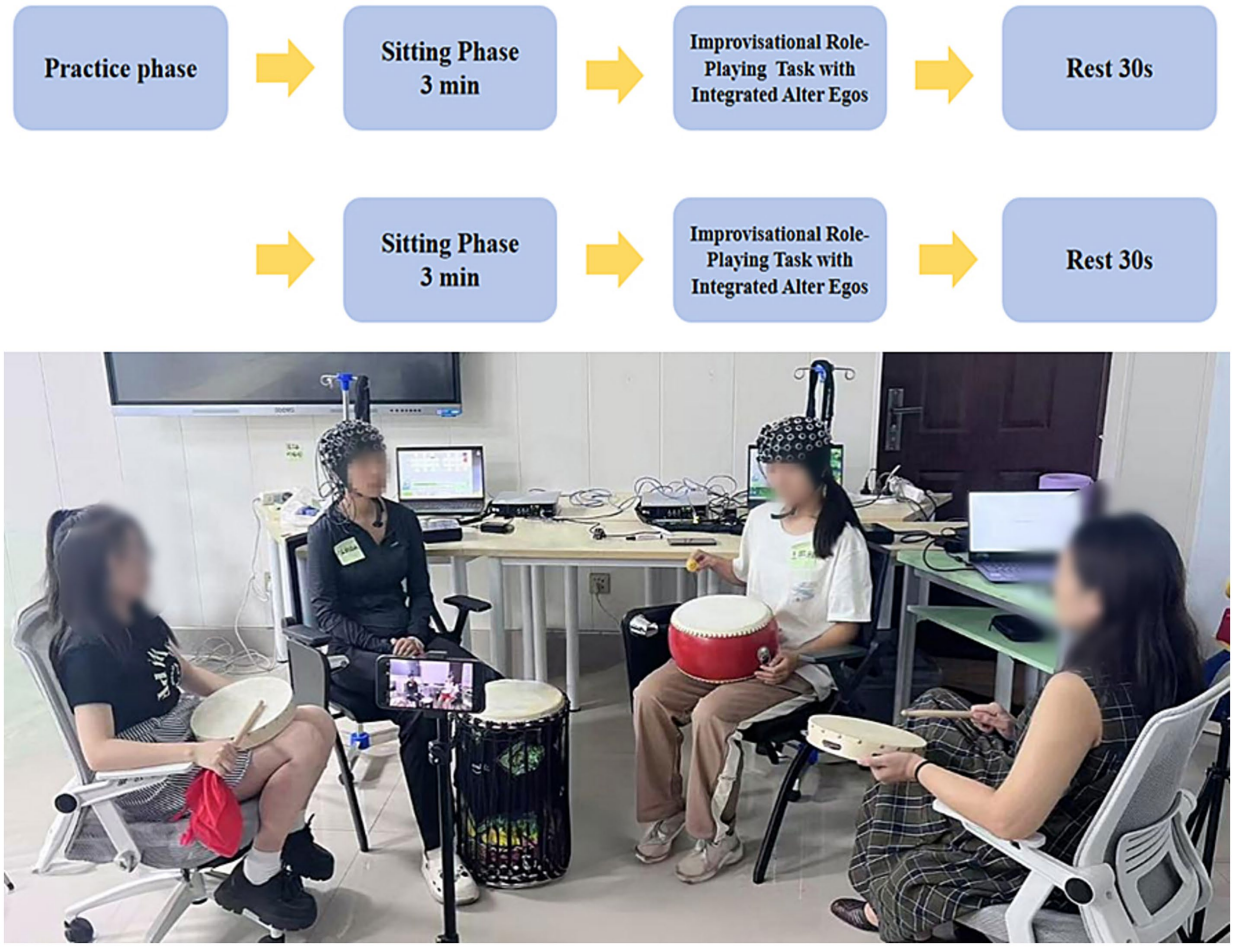
Schematic overview of the experimental design. The protocol proceeded sequentially through a practice phase, a resting phase, a formal experimental phase, and a 30-s rest interval, followed by one additional identical cycle. During the practice phase, the two participants completed a ~ 30-min joint rehearsal to become familiar with the theme and interaction format of improvisational role-playing. This was followed by a 3-min resting period, after which the formal experiment began. In each formal task block, the protagonist first performed musical improvisation, followed by verbal supplementation from the double; the auxiliary ego then performed musical improvisation, again followed by verbal supplementation from the double. Each task block lasted 3 min and was followed by a 30-s rest, after which the same task sequence was repeated once (the content within each block corresponded to the specific task requirements).

The standardized experimental procedure (see Figure 1) comprised the following phases:

(1) Pre-experiment Preparation:

After the theme and roles were determined, participants first completed open-ended written prompts to familiarize themselves with the scenario and their assigned role identities, followed by brief task training.

(2) Sitting Phase I (3 min):

Participants sat quietly with their eyes closed while maintaining neutral breathing.

(3) Task Phase I (3 min):

Role-playing and musical improvisation: Participant A (protagonist) initiated instrumental improvisation using free-form melody and rhythm without a predetermined score. Participant B (auxiliary ego) subsequently provided a musical response using the same or another available instrument, forming a bidirectional improvisational interaction. Two doubles stood approximately 45° behind the protagonist/auxiliary ego to provide verbal support, while the active role maintained continuous playing to ensure behavioral and contextual coherence.

Note: This phase constitutes the first 3 min of the total 6-min musical improvisation period.

Post–role-playing rest (30 s):

Participants sat quietly for a brief rest to disengage temporarily from the interactive state and return to a neutral baseline.

(4) Sitting Phase II (3 min):

Participants sat with their eyes open and avoided deliberate task-related thinking.

(5) Repeated Task Phase (3 min):

Role-playing and musical improvisation: Participant A again initiated free-form improvisation, followed by Participant B’s musical response. The two doubles remained in the same 45° positions and continued to provide verbal support, while instrumental playing was sustained to preserve the flow of interaction and expressive context.

Note: This phase comprises the remaining 3 min of musical improvisation, resulting in a total of 6 min across both task phases.

Post–role-playing rest (30 s):

Participants again engaged in a brief sitting rest to allow emotional and physiological states to stabilize.

(6) Emotion Ratings:

Participants rated their negative affect before and after the procedure using a 5-point Likert scale (1 = completely inconsistent, 5 = completely consistent). The entire experimental protocol lasted approximately 13 min, including sitting phases, task phases, repeated task phases, and brief rest periods.

The specific experimental procedure is illustrated in Figure 1.

### 2.3 fNIRS data acquisition

**Figure 2:**
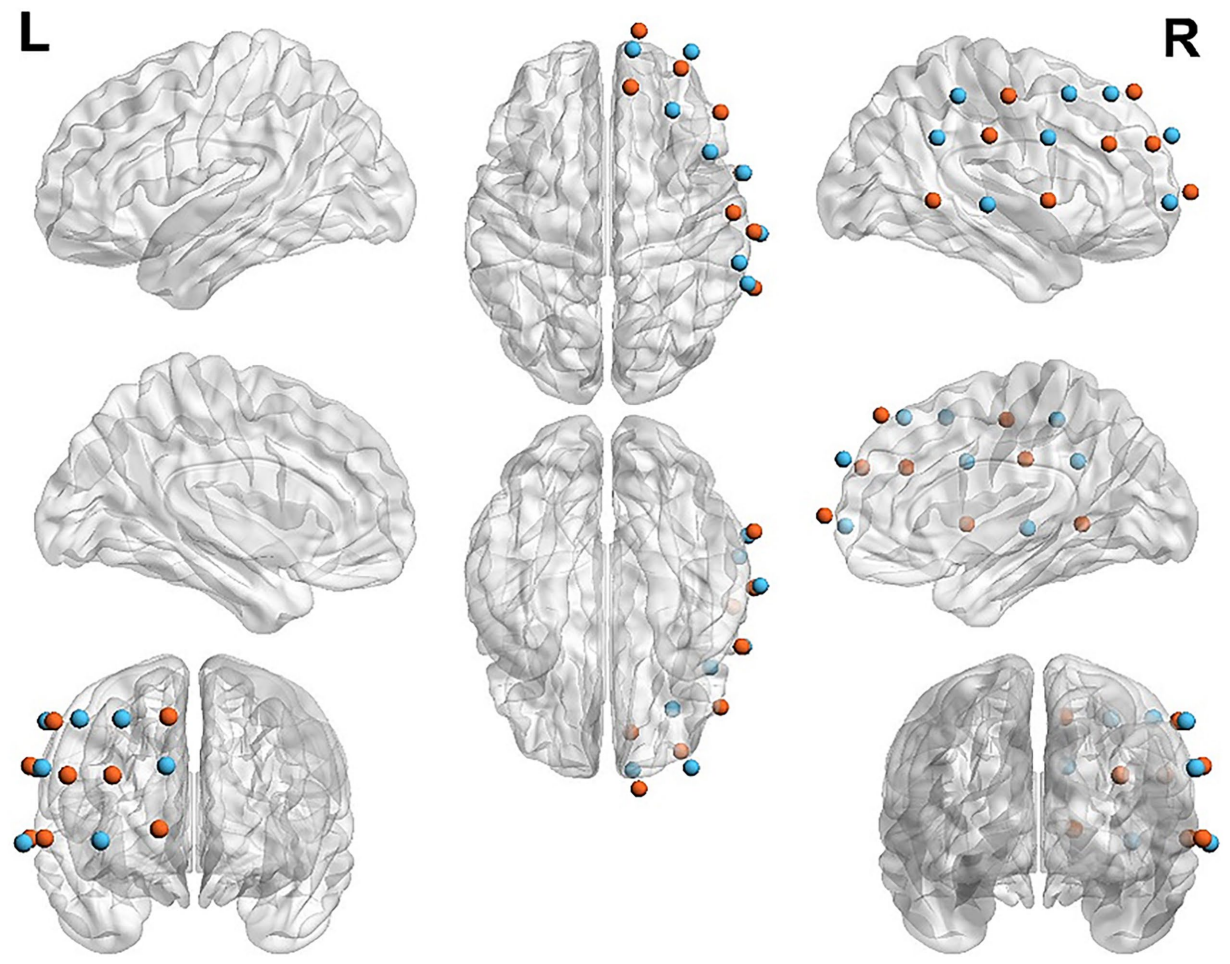
Brain region localization map. The map illustrates the regions of interest (ROIs), which are primarily concentrated in the R-PFC and the R-TPJ.

FNIRS data were acquired with a continuous-wave LIGHTNIRS system and kNIRS acquisition software (REV 0.5.400; Shimadzu Corporation, Japan). The system measures cortical hemodynamic activity at three wavelengths (780, 805, and 830 nm). Changes in oxygenated hemoglobin (HbO), deoxygenated hemoglobin (HbR), and total hemoglobin (HbT) concentrations were calculated based on the modified Beer–Lambert law (Cui et al., 2012). The sampling rate was 13.33 Hz. Each device included eight emitters and eight detectors, forming 20 measurement channels on the scalp. The source–detector separation was fixed at 3 cm, and each source–detector pair defined a measurement site corresponding to a specific cortical region. The channels primarily covered the right prefrontal cortex (PFC), right dorsolateral prefrontal cortex (DLPFC), and right temporoparietal junction (TPJ). Brain-region localization was performed in MATLAB R2023a. Regions of interest (ROIs) were first identified and the montage was planned using the fOLD toolbox (fNIRS Optodes’ Location Decider) in MATLAB (Zimeo Morais et al., 2018). Optode positions and channel layouts were then spatially co-registered to the standardized adult 3D head model embedded in the NIRS_SPM toolbox. Based on the source–detector geometry and optode coordinates, NIRS_SPM projected each channel onto the cortical surface and output the corresponding Montreal Neurological Institute (MNI) coordinates along with cortical localization probabilities for all 20 channels (De Witte et al., 2018; Zhang et al., 2018) (see Table 1 and Figure 2). NIRS_SPM further provides probabilistic cortical labels for each channel by integrating scalp-to-cortex sensitivity profiles with a standard anatomical atlas; to avoid over-interpretation of weak coverage, only regions with localization probabilities greater than 10% were reported (see Table 1).

**Table 1:** The MNI coordinates and probabilistic cortical localization of all 20 channels.

Channels	MNI x	MNI y	MNI z	Brodmann’s Area	*P*
CH01	12	59	40	9—Dorsolateral prefrontal cortex	0.82731
CH02	13	73	15	10—Frontopolar area	1
CH03	42	29	50	8—Includes Frontal eye fields	0.93886
CH04	20	46	50	8—Includes Frontal eye fields	0.91266
CH05	24	65	26	10—Frontopolar area	0.92015
CH06	28	69	10	10—Frontopolar area	1
CH07	52	28	39	9—Dorsolateral prefrontal cortex	0.67993
CH08	34	49	39	9—Dorsolateral prefrontal cortex	0.891
CH09	37	65	10	10—Frontopolar area	1
CH10	45	49	15	10—Frontopolar area	0.53648
CH11	64	−64	6	6—Pre-Motor and Supplementary Motor Cortex	0.97193
CH12	66	8	19	6—Pre-Motor and Supplementary Motor Cortex	0.45307
CH13	70	−35	19	40—Supramarginal gyrus part of Wernicke’s area	0.9125
CH14	65	−29	50	40—Supramarginal gyrus part of Wernicke’s area	0.45353
CH15	69	−9	31	6—Pre-Motor and Supplementary Motor Cortex	0.61489
CH16	71	−10	0	21—Middle Temporal gyrus	0.55882
CH17	69	−49	13	22—Superior Temporal gyrus	0.82026
CH18	61	−38	40	40—Supramarginal gyrus part of Wernicke’s area	0.65116
CH19	72	−22	42	42—Primary and Auditory Association Cortex	0.63125
CH20	73	−35	21	21—Middle Temporal gyrus	0.59621

The corresponding Brodmann areas were identified using a brain atlas. Individual channels could cover multiple brain regions, with the sum of probabilities for overlapping areas not exceeding 1. Only channels covering more than 10% of a brain area were reported.

Our emphasis on right-hemispheric regions was based on three factors. First, a growing body of evidence suggests that social–emotional functions show a relative right-hemisphere predominance, encompassing interpersonal interaction, decoding of nonverbal signals, emotional experience and regulation, and empathic processing (Zhang et al., 2020; Xu et al., 2023). Second, because the number of fNIRS channels is inherently limited, allocating optodes to theoretically prioritized right-hemisphere sites maximizes coverage of relevant circuitry and improves measurement efficiency. Third, limiting statistical tests to the right hemisphere reduces the multiplicity burden, which in turn enhances sensitivity under stringent corrections by lowering the likelihood of Type II errors and increasing the probability of detecting task-evoked changes in intra-brain activation and/or inter-brain synchrony within the predefined targets.

To control for movement-related noise, optodes were firmly secured with an elastic cap and sponge padding, and participants were reminded to remain as still as possible during recording. Signal quality was continuously checked online by the experimenter throughout acquisition. In preprocessing, motion-contaminated epochs were automatically detected using derivative- and amplitude-based criteria and were subsequently corrected via the Correlation-Based Signal Improvement (CBSI) approach. Channels showing consistently low signal-to-noise ratios or residual artifacts after correction were removed prior to statistical analyses.

### 2.4 Data analysis

#### 2.4.1 Behavioral data analysis

Participants completed both the Negative Emotion Questionnaire and the Visual Analogue Scale (commonly used in short-term intervention studies to assess pre-post changes in anxiety) before and after the music psychodrama intervention. Independent-samples *t*-tests were conducted to compare pre- and post-test scores, in order to determine whether the intervention effectively alleviated negative emotions.

#### 2.4.2 fNIRS data analysis

Preprocessing: Raw fNIRS recordings were preprocessed in MATLAB (R2024a) using the NIRS-KIT toolbox (Hou et al., 2021). Data quality was first evaluated by visually inspecting the wavelet spectrum for a distinct cardiac component around ~1 Hz; channels lacking this feature were marked as invalid (Nguyen et al., 2020). Overall, 96.576% of channels met the quality criterion and were retained. Dyads were excluded only when more than 50% of their channels were invalid (Lu et al., 2020); no dyads met this exclusion rule. Next, optical density signals from all measurement channels were converted to HbO/HbR concentration changes via the modified Beer–Lambert law implemented in NIRS-KIT (Hou et al., 2021). To correct baseline drift and motion-related contamination, polynomial detrending was applied to remove linear or nonlinear trends, followed by Correlation-Based Signal Improvement (CBSI) (Cui et al., 2010). In CBSI, the scaling factor was automatically computed for each channel as *α* = σ_HbO / σ_HbR to restore the intrinsic negative HbO–HbR correlation. Motion correction was performed separately for each dyad to preserve within-pair signal structure. Finally, systemic and environmental noise was reduced using global-signal (noise) regression to attenuate scalp blood-flow components, and a temporal band-pass filter of 0.01–0.20 Hz was applied to retain task-related fluctuations while removing slow drifts and high-frequency physiological noise (Emberson et al., 2016; Yin et al., 2025).

Intra-brain activation: We focused on the analysis of oxygenated hemoglobin (HbO) signals, as they are more directly associated with cognitive activation compared to deoxygenated hemoglobin (HbR) signals (Zhang et al., 2024). A general linear model (GLM) was then applied to model HbO signal changes for each participant. Specifically, the task duration was convolved with a standard hemodynamic response function (HRF) to construct the regression model in the GLM, and *β*-values were extracted for each condition (Wang et al., 2024). Finally, independent-sample t-tests were performed on the *β*-values obtained from the role-playing and resting conditions to evaluate condition-related differences in intra-brain activation, and all comparisons were corrected for multiple comparisons using the false discovery rate (FDR) method.

**Figure 3:**
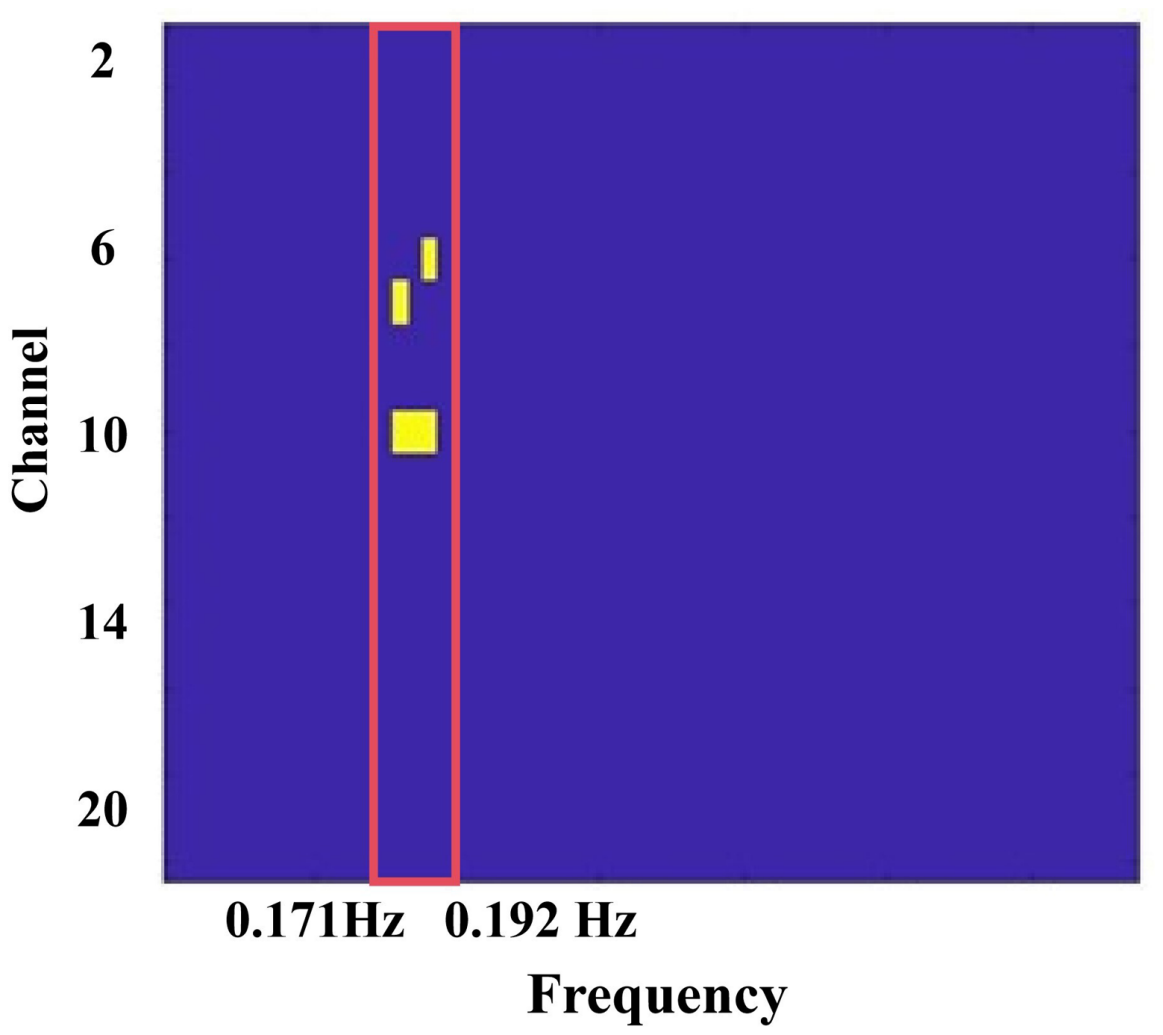
Fdr-corrected *p*-value map for task type. IBS during the role-playing task was significantly higher than that during the resting-state condition, with a significant frequency range identified between 0.171 and 0.192 Hz. The yellow squares indicate the frequency band of interest (FOI).

Inter-brain Synchrony: The unfiltered preprocessed data were imported into MATLAB 2023a, and inter-brain coherence values were calculated using the Wavelet Transform Coherence (WTC) toolbox (Grinsted et al., 2004). WTC was used to compute wavelet coherence scores for 20 paired channels between participants. This method assesses the temporal relationship of HbO signals between each pair of participants (Grinsted et al., 2004). After obtaining the wavelet coherence values, we applied a Fisher Z transformation (Fisher *Z*-statistics) to the data. Subsequently, a data-driven frequency band selection method was employed to compare WTC values between the two task conditions (Yin et al., 2025). Independent-samples t-tests were then performed on the WTC values, with task type (role-playing vs. resting) as the independent variable. A significant difference was observed in the frequency range of 0.171–0.192 Hz, which was identified as the frequency of interest (FOI), as shown in Figure 3. This selection was further validated using a *post hoc* FDR correction. This frequency band effectively excluded high- and low-frequency physiological noise such as blood pressure fluctuations (~0.1 Hz), respiration (~0.2–0.3 Hz), and heart rate (~1 Hz), as well as other related physiological artifacts (Nozawa et al., 2016).

## 3 Results

### 3.1 Behavioral results

An independent-samples *t*-test was performed on both the Negative Emotion Questionnaire and the Visual Analogue Scale scores, revealing a significant main effect of time (negative emotion: *t* = 3.417, *p* = 0.001; anxiety: *t* = 2.655, *p* = 0.009). Follow-up analyses showed that both negative emotion and anxiety scores were significantly higher at pre-test compared with post-test (negative emotion: *p* = 0.001; anxiety: *p* = 0.009).

### 3.2 Intra-brain activation results

**Figure 4:**
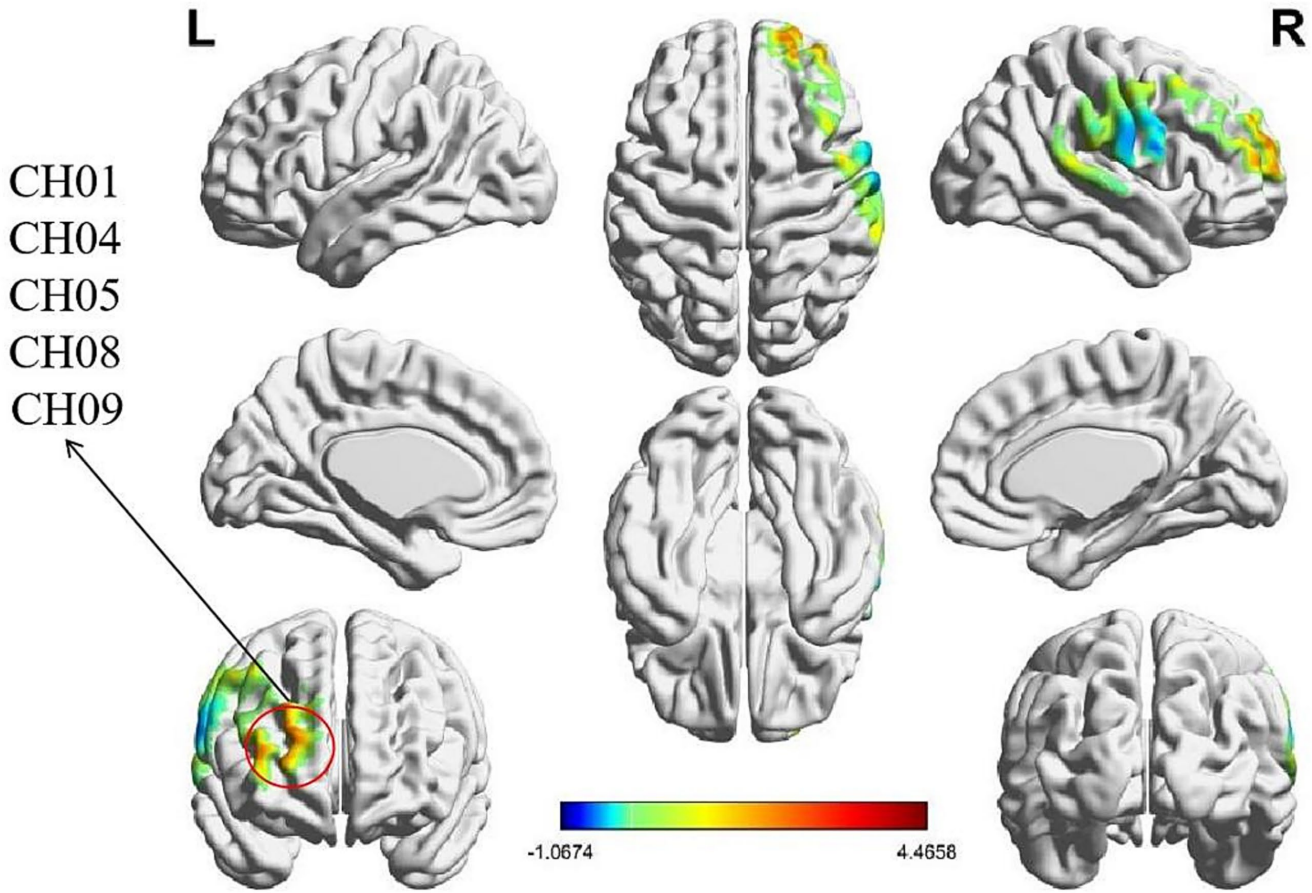
Heatmap of intra-brain activation by task type. Intra-brain activation during the role-playing task was significantly higher than during the resting-state condition, with significance defined at an FDR-corrected threshold of *p* < 0.05 (i.e., *q* < 0.05). Red regions denote the significant channels: CH01, CH04, CH05, CH08, and CH09.

Independent-samples *t*-tests were conducted to examine the effect of task type on intra-brain activation values across 20 channels. Significant main effects of task type were observed in the following channels: CH01 and CH08 (R-DLPFC), CH04 (right frontal eye fields, FEF), and CH05 and CH09 (Right frontopolar area, R-FT). Significant differences were found as follows: CH01: *t* = 3.457, *p* = 0.008, Cohen’s d = 0.360; CH04: *t* = 4.466, *p* < 0.001, Cohen’s d = 0.466; CH05: *t* = 2.847, *p* = 0.027, Cohen’s d = 0.297; CH08: *t* = 2.697, *p* = 0.033, Cohen’s d = 0.281; CH09: *t* = 2.955, *p* = 0.027, Cohen’s d = 0.308. *Post hoc* analyses further indicated that intra-brain activation during the role-playing task was significantly higher than during the resting-state task across these channels (CH01: *p* = 0.008; CH04: *p* < 0.001; CH05: *p* = 0.027; CH08: *p* = 0.033; CH09: *p* = 0.027). The corresponding intra-brain activation heatmaps are presented in Figure 4.

### 3.3 Inter-brain synchrony results

**Figure 5:**
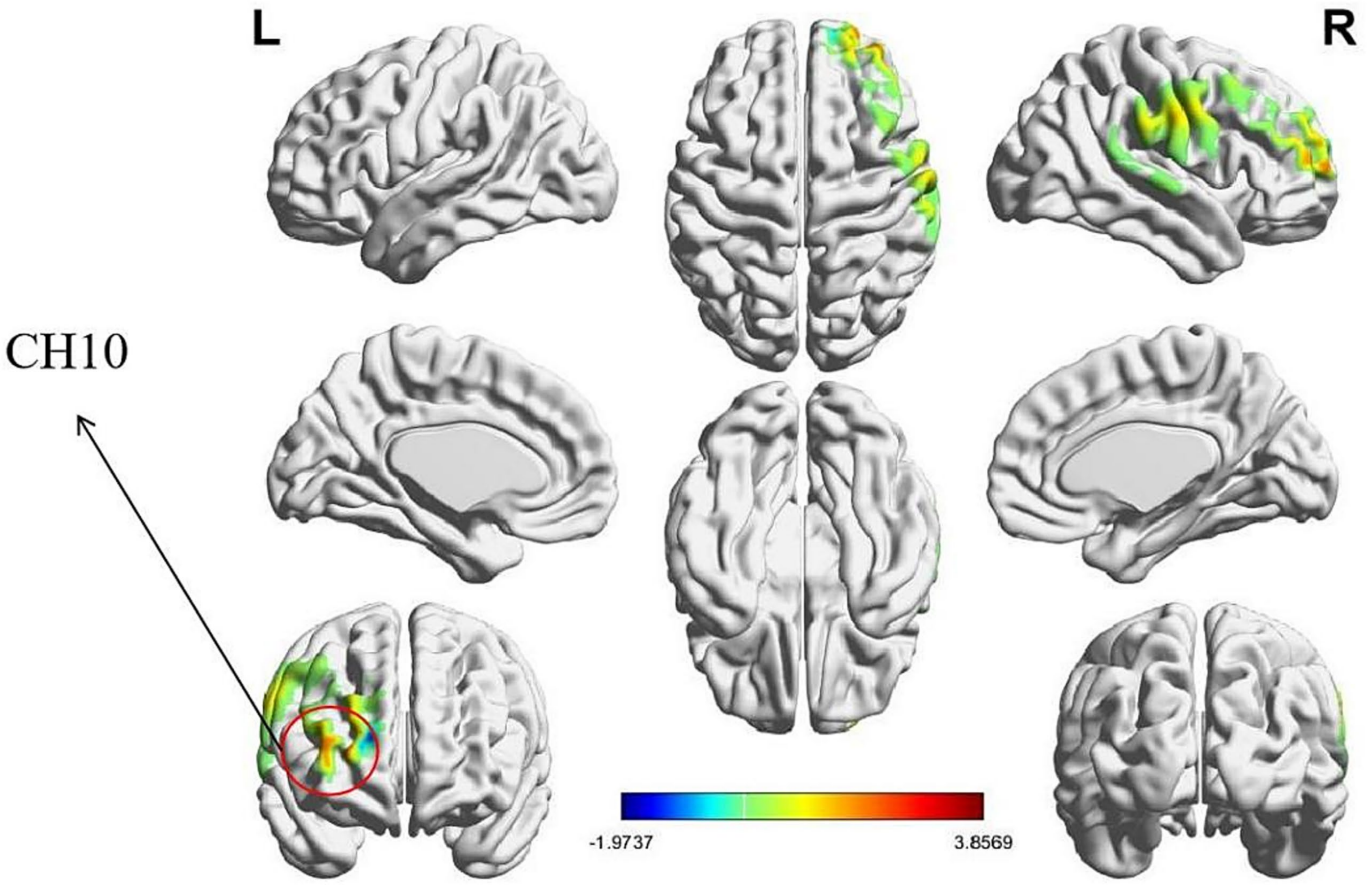
IBS heatmap by task type. IBS during the role-playing task was significantly higher than during the resting-state condition, with significance defined at an FDR-corrected threshold of *q* < 0.05 (*p* < 0.05). The red region denotes the significant channel (CH10).

Independent-samples t-tests were conducted to examine the effect of task type on IBS values across 20 channels. The results revealed significant main effects of task type in the following channels: CH10 (R-FT). Significant differences were observed as follows: CH10: *t* = 3.857, *p* = 0.007, Cohen’s *d* = 0.402. Post hoc analyses indicated that IBS values during the role-playing task were significantly higher than those during the resting-state task in this channel (CH10: *p* = 0.007). The results of IBS heatmaps are presented in Figure 5.

## 4 Discussion

Although previous studies have examined brain activation and IBS during psychodrama role-playing, the neural activity patterns and interpersonal neural coupling elicited by improvisational music psychodrama may differ substantially from those observed in conventional psychodrama. Therefore, the neural mechanisms underlying music psychodrama and its effects on negative emotions require further in-depth investigation. To address this gap, the present study employed an improvisational role-playing paradigm incorporating the doubling technique, combined with fNIRS hyperscanning, to examine the effects of music psychodrama on negative emotions and its associated neural mechanisms.

The results showed that after participating in music psychodrama role-playing, individuals exhibited a significant reduction in negative emotions. At the neural level, enhanced activation was observed in the R-DLPFC and the R-FT region, along with a significant increase in inter-brain synchrony within the Right FT region. These findings suggest that music psychodrama may alleviate negative emotions by engaging key brain regions involved in cognitive control and emotional regulation—particularly the R-DLPFC and R-FT—which have been consistently shown in previous research to play essential roles in the perception and regulation of negative affect. This interpretation aligns with extensive empirical evidence highlighting the functional contributions of the R-DLPFC and R-FT to negative emotion processing and further indicates that music psychodrama can enhance interpersonal neural synchrony (FT-IBS) between participants (Kaffenberger et al., 2008; Wang S. K. et al., 2025; Wang Y. et al., 2025; Cheng et al., 2024a). Taken together, these findings elucidate the potential neural mechanisms underlying the therapeutic effects of music psychodrama and provide robust scientific support for its application as an evidence-based psychological intervention.

The core technique of music psychodrama—musical improvisation—has been widely recognized for its distinctive benefits in specific clinical populations. These benefits include mitigating neurological impairments, alleviating mental health symptoms, reducing stress and anxiety, and improving communication and joint-attention behaviors in children with autism spectrum disorders. Role-playing facilitates the expression of emotions that are difficult to articulate verbally and promotes cognitive restructuring. Its principal advantages lie in providing participants with a safe and controllable setting for practice, minimizing real-world risks, and offering structured opportunities for reflection and feedback (Liberali and Grosseman, 2015). By fostering decentering, role-playing enables individuals to establish psychological distance from stressful or traumatic experiences, thereby promoting emotional release through physical movement and embodied role immersion, which can reduce anxiety, depression, and other negative emotions (Singla et al., 2023). Moreover, prior research suggests that musical improvisation exerts its effects through several key mechanisms, including the integration of conscious and unconscious processes, the attentional engagement required to enter a creative “flow” state, and the capacity to express complex or suppressed emotions without reliance on verbal articulation (MacDonald and Wilson, 2014). In addition, when the double observes and imitates the participant’s actions, the mirror neuron system is activated. This activation resembles the motor activation that occurs when the observer plans and executes the same action. Because the two activations are similar, the observer understands the intention behind others’ actions directly, without explicit inference (Sinigaglia and Rizzolatti, 2011). By enhancing emotional resonance between participants and their doubles, this mechanism strengthens perceived social support, reduces loneliness, and consequently alleviates negative emotions. Therefore, by providing novel experimental evidence, the present study addresses an important gap in the literature and confirms Hypothesis 1: The results indicate that role-playing–based music psychodrama is associated with a significant reduction in negative affect, suggesting its potential value as an intervention for emotion regulation and psychological support.

This study yielded two main findings. First, individuals who engaged in music psychodrama role-playing showed stronger intra-brain activation in the R-FT region and the R-DLPFC. This pattern is consistent with prior work on psychodramatic mechanisms, demonstrating that role-playing recruits prefrontal systems and that adopting a first-person perspective (i.e., “imagining being person X”) elicits greater engagement of the ventromedial prefrontal cortex than a third-person perspective (Kipper and Ritchie, 2003; Goldstein, 1967). Moreover, a substantial body of research links the DLPFC to goal representation and maintenance as well as the allocation of attentional resources. Accordingly, as a key node of the executive-control network, the DLPFC supports the retention of role-related goals and information in working memory, the dynamic deployment of attention to flexibly meet improvisational demands, and the top–down, deliberate regulation of emotional responses (Snitz et al., 2005). In addition, the frontotemporal system—particularly the frontopolar cortex—has been implicated in prospective memory regulation, multitasking, relational integration, internal-state processing, and self-referential evaluation. Rather than issuing direct executive commands, the FT region facilitates understanding of characters’ emotions and intentions and promotes affective resonance (empathy) with interaction partners (e.g., doubles) during role-playing and musical improvisation, thereby supporting its socio-emotional functions (Hoffmann and Bar-On, 2012). Converging neuroimaging evidence on musical improvisation further indicates that improvisational performance strengthens functional connectivity within emotion- and reward-related networks while modulating activity in the PFC and TPJ (Limb and Braun, 2008; Beaty, 2015; Sasaki et al., 2019).

However, although Hypothesis 2 predicted prominent TPJ involvement, the significant effects observed in the present study were concentrated in R-FT and R-DLPFC channels rather than R-TPJ. This pattern may reflect the stronger optical sensitivity and statistical power of frontal/frontotemporal fNIRS channels relative to temporoparietal sites, given that fNIRS sensitivity is constrained by optode geometry, scalp–cortex distance, and inter-individual variability in hair/skin coupling. In addition, the improvisational role-playing paradigm incorporating doubling places substantial demands on cognitive control, working memory maintenance, and flexible regulation of negative affect—functions primarily supported by DLPFC and broader frontotemporal circuitry. Therefore, TPJ-related mentalizing processes may have been engaged in a more distributed manner within a wider frontotemporal social-cognitive network and/or remained below corrected significance due to limited TPJ sampling and statistical power.

The second key finding of this study is that music psychodrama role-playing significantly enhanced IBS in the R-FT region. This result indicates a higher degree of neural alignment between interaction partners during socio-emotional and communicative processes, consistent with the view that IBS serves as a quantitative neural index of interpersonal coordination and affective resonance. From a broader social–emotional network perspective, right-hemispheric fronto-temporal and temporoparietal systems support the integrative processing of affective and expressive cues that provides an essential cognitive foundation for emotional expression (LaVarco et al., 2022). Accordingly, the increase in IBS within the R-FT region can be interpreted as reflecting tighter cross-brain coupling during shared musical–dramatic engagement (Cui et al., 2012). Previous research has demonstrated that IBS reliably emerges across diverse musical interaction contexts and depends on a distributed neural network encompassing frontal, parietal, and temporal cortices, which plays a central role in interpersonal coordination (Cheng et al., 2024b). In addition, the FT has been proposed to function as a stage-dependent hub in action–intention understanding, characterized by a transition from bottom-up action matching to top-down intention inference through interactions with mentalizing-related networks (Zhang et al., 2025). Taken together, these findings support Hypothesis 2 and further highlight the value of hyperscanning for elucidating the neural foundations of multi-person interaction in music psychodrama.

This study still has several limitations that should be addressed in future research. First, given the inherent spatial-resolution constraints of fNIRS, the present work could only localize cortical activity at a relatively macroscopic level; combining fNIRS with EEG hyperscanning in subsequent studies would substantially improve both spatial and temporal precision, enabling a more fine-grained characterization of dynamic neural processes during interaction. Second, due to restricted optode coverage, measurements were limited to the right prefrontal and temporoparietal regions; future investigations should adopt bilateral or high-density fNIRS montages to achieve more comprehensive whole-brain coverage and network-level evidence. Third, because no control condition was included, active control groups are needed to establish the specificity and causal nature of the intervention effects. In addition, the absence of physiological monitoring suggests that future studies should integrate multimodal assessments—such as respiratory and heart-rate measures as well as automated facial-expression analysis—to capture emotional responses more comprehensively. Moreover, the sample consisted exclusively of Chinese university students with elevated social anxiety traits, which limits the generalizability of the findings to other populations, age groups, and cultural contexts. Finally, the current sample size was relatively small and the gender distribution was homogeneous; future work should expand recruitment and increase sample heterogeneity by including a wider range of dyad types (e.g., parent–child, teacher–student, supervisor–subordinate pairs) and systematically testing demographic moderators such as gender. Larger samples will also allow meaningful subgroup analyses and direct comparisons between music-assisted psychodrama and conventional verbal psychodrama, thereby clarifying their distinct neurobehavioral mechanisms.

## 5 Conclusion

This study employed fNIRS-based hyperscanning to investigate the neural mechanisms through which role-playing techniques in music psychodrama alleviate negative emotions. The findings revealed that participants who engaged in music psychodrama role-playing experienced significant reductions in negative emotions. Moreover, they exhibited enhanced intra-brain activation in the R-FT and R-DLPFC, as well as increased IBS in the R-FT. These results deepen our understanding of the neurobiological mechanisms underlying music psychodrama interactions and their effects on negative affect. Importantly, the findings also suggest that IBS may serve as a potential neural marker of therapeutic processes in psychodrama, providing novel directions for future research on the mechanisms of music psychodrama interventions.

## Data Availability

The raw data supporting the conclusions of this article will be made available by the authors, without undue reservation.
